# Kidney and Urinary Tract Involvement in Kawasaki Disease

**DOI:** 10.1155/2013/831834

**Published:** 2013-10-31

**Authors:** Toru Watanabe

**Affiliations:** Department of Pediatrics, Niigata City General Hospital, 463-7 Shumoku, Chuo-ku, Niigata City 950-1197, Japan

## Abstract

Kawasaki disease (KD) is a systemic vasculitis and can develop multiple organ injuries including kidney and urinary tract involvement. These disorders include pyuria, prerenal acute kidney injury (AKI), renal AKI caused by tubulointerstitial nephritis (TIN), hemolytic uremic syndrome (HUS), and immune-complex mediated nephropathy, renal AKI associated with either Kawasaki disease shock syndrome or unknown causes, acute nephritic syndrome (ANS), nephrotic syndrome (NS), renal tubular abnormalities, renal abnormalities in imaging studies, and renal artery lesions (aneurysms and stenosis). Pyuria is common in KD and originates from the urethra and/or the kidney. TIN with AKI and renal tubular abnormalities probably result from renal parenchymal inflammation caused by T-cell activation. HUS and renal artery lesions are caused by vascular endothelial injuries resulting from vasculitis. Some patients with ANS have immunological abnormalities associated with immune-complex formation. Nephromegaly and renal parenchymal inflammatory foci are detected frequently in patients with KD by renal ultrasonography and renal scintigraphy, respectively. Although the precise pathogenesis of KD is not completely understood, renal vasculitis, immune-complex mediated kidney injuries, or T-cell immune-regulatory abnormalities have been proposed as possible mechanisms for the development of kidney and urinary tract injuries.

## 1. Introduction

Kawasaki disease (KD) is an acute febrile vasculitis that predominantly affects children ≤5 year of age [1, 2]. Children with KD typically have an acute onset of fever followed by signs of mucosal inflammation and vasodilatation that evolve over the first week of the illness. Laboratory tests reveal a marked systemic inflammatory response [3]. KD is the leading cause of acquired heart disease in children in most developed countries, including Japan and the USA [4, 5]. KD predominantly causes vasculitis in medium-sized arteries, with a striking predilection for the coronary arteries [2, 4]. The earliest pathological change in the vessel wall is edema of endothelial and smooth muscle cells with intense inflammatory infiltration of the vascular wall, initially by polymorphonuclear cells and rapidly thereafter by macrophages, lymphocytes (primarily CD8+ T cells), and plasma cells [4]. 

The cause of KD remains unknown, but it is thought that the immune system is activated by an infectious trigger in genetically susceptible hosts [5]. Although there is considerable controversy regarding the mechanism of immune system activation, recent data suggested that T-cell activation is important in determining the susceptibility and severity of KD [5]. Furthermore, susceptibility genes for KD have been identified by successive studies using a genome-wide approach. These include the inositol 1,4,5-triphosphate kinase-C (*ITPKC*), caspase-3 (*CASP3*), CD40 ligand (*CD40L*), *FAM167A*-*BLK*, the Fc fragment of IgG, low affinity IIa, receptor (*FCGR2A*), and the human leukocyte antigen (HLA) genes [6]. 

 Because KD is a systemic vasculitis, multiple organ involvement can develop, including coronary artery lesions (CALs), carditis, arthritis, hepatitis, central nervous system (CNS) disease [3, 7], KD shock syndrome (KDSS) [8], muscle involvement [7, 9], hyponatremia [10–12], and kidney and urinary tract involvement.

With the exception of sterile pyuria and trace proteinuria, kidney and urinary tract involvement in KD is uncommon [1, 3, 13]. However, as shown in the paper, such complications are increasingly being reported, including pyuria, prerenal acute kidney injury (AKI), renal AKI caused by tubulointerstitial nephritis (TIN), hemolytic uremic syndrome (HUS), immune-complex mediated nephropathy, renal AKI associated with KDSS or unknown causes, acute nephritic syndrome (ANS), nephrotic syndrome (NS), renal tubular abnormalities, renal abnormalities in imaging studies, and renal artery lesions. Because renal complications can contribute to an acute or chronic deterioration in the condition of patients, it is important to clarify the clinical features and pathogenesis of renal involvement in patients with KD. The following is a review of kidney and urinary tract involvement in KD.

## 2. Pyuria 

Patients with KD often present with abnormal urinary findings, including proteinuria, hematuria, and pyuria [1, 12, 13]. Of these, pyuria is the most common abnormal finding [12, 14, 15], with some patients with KD and pyuria being misdiagnosed as having acute pyelonephritis [16, 17]. In a previous study, we showed 43.5% of patients with KD had sterile pyuria, a proportion similar to that reported by Melish et al. [13], Wirojanan et al. [18], Barone et al. [19], and Shike et al. [20] of 62.5%, 33%, 50%, and 74%, respectively. On the other hand, Turner and Coulthard [21] reported that 43% of febrile children without urinary tract infection had moderate pyuria (10–100 cells/high-power field) with only 9% of febrile children having obvious pyuria (>100 cells/high-power field). This finding indicated that pyuria may be a nonspecific feature of fever in acute childhood illness. However, because one-third of patients with KD exhibited obvious pyuria in our study [14], we considered pyuria was a specific feature of the disease.

Sterile pyuria in KD is associated with mononuclear cells in the urine [3]. Ohta et al. [22] demonstrated that the majority of cells in urine specimens of KD patients with pyuria were mononuclear cells, but not neutrophils. Kobayashi et al. [23] also showed that mononuclear cells with intracytoplasmic inclusion were present in the urinary sediments of a patient with KD. 

Sterile pyuria in KD is thought to be due to urethritis caused by a non-specific vasculitis of the urethra [3]. Melish et al. [13] reported four KD patients with sterile pyuria in voided urine samples who did not have leukocytes in bladder urine obtained by bladder taps. This suggested that urethral inflammation was the source of the urinary leukocytes. However, we reported that 5 of 10 KD patients with sterile pyuria in voided urine samples also had leukocytes in bladder urine, with higher levels of urinary protein and *β*2-microglobulin and serum blood urea nitrogen and creatinine concentrations than patients without leukocytes in their bladder samples or patients without pyuria in voided urine [14]. These results suggest that some patients with KD develop sterile pyuria that originates from the urethra and/or the kidney as a result of mild and subclinical renal injury [14]. In addition, we recently reported a case of acute cystitis in a patient with KD, which suggested sterile pyuria in KD may originate from the bladder due to cystitis [24].

 Pyuria is not always sterile in patients with KD. Shiono et al. [25] reported a KD patient with a left vesicoureteral reflux associated with pyuria and pyelonephritis due to *Escherichia coli*. Wu et al. [26] reported that 8 of 75 patients with KD had bacterial pyuria, while Benseler et al. [27] reported that 42 of 129 (33%) patients with KD had ≥1 confirmed bacterial or viral infection at diagnosis, with 4 (3%) of these patients having a urinary tract infection (UTI). No difference in clinical phenotype or coronary artery outcome was observed in patients with a UTI compared with those without a UTI [26, 27]. It remains unclear whether bacterial UTI induces an immune response in patients with KD or whether this occurs only when the patients have a coexisting infection [26]. However, because the immune system is thought to be activated by an infectious trigger in genetically susceptible hosts with KD [5], a UTI may induce clinical features typical of KD.

## 3. Acute Kidney Injury

AKI is the new consensus term for acute renal failure (ARF) and has replaced ARF in order to emphasize that a continuum of kidney injury exists that begins long before sufficient loss of excretory kidney function can be measured by standard laboratory tests [28]. 

 AKI is usually divided into three broad pathophysiologic categories according to the cause: prerenal AKI, characterized by decreased kidney perfusion in which there is no parenchymal damage to the kidney, renal AKI, which results from the renal parenchyma injuries, or postrenal AKI, caused by acute obstruction of the urinary tract [28]. 

In patients with KD, both prerenal and renal AKI have been reported. While TIN, HUS, immune-complex mediated nephropathy, and KDSS have been reported as causes of renal AKI, the cause of renal injury has not been established in other patients. 

### 3.1. Prerenal Acute Kidney Injury

Prerenal AKI has been reported in three patients with KD. Nakanishi et al. [29] reported a 12-year-old boy with KD who developed prerenal AKI and acute heart failure (AHF). A renal biopsy demonstrated no abnormal findings in the glomeruli or tubulointerstitium. Senzaki et al. [30] reported an 8-year-old boy with KD who developed prerenal AKI and AHF, while Adachi [31] described a 2-year-old girl with KD who developed prerenal AKI caused by hypovolemia due to gastrointestinal losses and AHF. All three patients had AHF concomitant with AKI with their renal function recovering completely following improvement of AHF. Because AHF causes renal hypoperfusion due to decreased cardiac output [32], AHF may play a central role in the development of prerenal AKI in patients with KD.

### 3.2. Renal Acute Kidney Injury

#### 3.2.1. Tubulointerstitial Nephritis

TIN is a frequent cause of AKI and is characterized by the presence of an inflammatory cell infiltrate in the kidney interstitium [33]. Some cases of TIN are secondary to diseases with immune-mediated mechanisms such as systemic lupus erythematosus, Sjögren's disease, or vasculitis [33]. The presence of T-helper and T-suppressor lymphocytes in the cellular infiltrates suggests that these cells have a pathogenic role in TIN [33]. 

AKI due to TIN has been reported in five patients with KD. Veiga et al. [34] reported a 2-year-old boy with KD who developed AKI due to TIN. A renal biopsy revealed diffuse interstitial infiltration of mononuclear and polymorphonuclear leukocytes. The patient recovered with supportive care alone. Kawamura [35] reported a 9-year-old girl with KD who developed AKI with a renal biopsy showing mild interstitial mononuclear infiltration. The patient underwent hemodialysis and recovered without renal sequelae but with CALs. Ashida et al. [36] reported a 5-year-old boy with KD who developed AKI due to TIN. The patient recovered with supportive care alone. Bonany et al. reported [37] an 8-year-old boy with KD who developed AKI and nephrotic syndrome, in whom renal biopsy showed TIN with mild mesangial expansion and no vessel involvement. The patient recovered with supportive care alone. Papadodima et al. [38] examined the kidney autopsy findings of an 11-year-old boy who died of coronary arterial thrombosis associated with KD and found that the patient had TIN with mild expansion of the mesangial matrix.

Further evidence was reported by Ogawa [39] who examined kidney specimens from 25 patients who died from KD and showed that these patients frequently had interstitial leukocytes infiltrations (36%), especially in patients with duration of illness <50 days (100%). Asaji et al. also reported that 25% of patients who died from KD had interstitial infiltration of leukocytes [40]. These findings suggest that TIN may occur more commonly in KD patients. Because T cell activation contributes to the development of TIN [33] and KD [5], it may also cause TIN in patients with KD.

However, it is necessary to rule out *Y. pseudotuberculosis* infection in patients with KD and AKI caused by TIN [41]. *Y. pseudotuberculosis* is a gram-negative coccobacillus that causes diarrhea, abdominal pain, fever, skin rash, conjunctivitis, erythema nodosum, and lymphadenopathy [42, 43]. Typical KD-like findings developed in 8.8%–10.8% of patients with *Y. pseudotuberculosis* infections [43, 44], while, AKI occurred in 9.6%–13.6% of these patients [43, 45]. In addition, the renal histology of nearly all patients with *Y. pseudotuberculosis* infection who underwent renal biopsy showed TIN [43, 45–47]. Therefore, AKI is more likely to develop in patients with *Y. pseudotuberculosis* infection, which mimics KD, than in patients with true KD. 

#### 3.2.2. Hemolytic Uremic Syndrome

HUS is a disease characterized by nonimmune hemolytic anemia, thrombocytopenia, and renal impairment [48, 49]. Microvascular injury with endothelial cell damage is a pathological characteristic of all forms of HUS [49]. The various etiologies of HUS allow classification into infection-induced, genetic, medication-induced, and HUS associated with systemic diseases characterized by microvascular injury [49].

 HUS has been reported in only two patients with KD and AKI [50, 51]. Ferriero and Wolfsdorf [50] reported a 2-year-old girl with KD who had clinical and laboratory features of mild HUS, who recovered with supportive care alone. Heldrich et al. [51] reported a 3-year-old girl with KD who developed HUS and Henoch-Schönlein purpura, which required adjustments in therapy. 

Although the pathogenesis underlying the development of HUS in both patients was unclear because they did not undergo renal biopsy, it is possible that the vasculitis associated with KD may have involved the kidney producing endothelial injuries in the renal and glomerular endothelium, leading to a clinical picture of HUS [50].

#### 3.2.3. Immune-Complex Mediated Nephropathy

Nagamatsu et al. [52] reported renal histologic findings of a 3-year-old boy with KD who developed renal AKI. Although light microscopy showed that nearly all the glomeruli were normal, electron microscopy revealed electron dense deposits in the subepithelial spaces and in the podocytes. This suggested the possibility of glomerular derangement by immune complex in this patient. 

#### 3.2.4. Renal Acute Kidney Injury Associated with Kawasaki Disease Shock Syndrome

KDSS has recently been proposed as severe form of KD characterized by systolic hypotension or clinical signs of poor perfusion [8, 53]. KDSS is associated with more severe changes in laboratory inflammatory markers and greater risk of coronary artery abnormalities, mitral regurgitation, and prolonged myocardial dysfunction [8]. 

Gatterre et al. [54] studied 11 patients with KDSS and reported that 10 of these patients developed AKI, with 8 of these patients having multiple organ dysfunction syndrome (MODS). All 10 patients recovered from AKI without any renal sequels. Mac Ardle et al. [55] reported a 2-year-old boy with clinical features of KDSS who developed AKI that required peritoneal dialysis. A renal biopsy showed normal glomeruli and a patchy immune-type infiltrate that contained plasma cells and eosinophils, with evidence of recovering acute tubular necrosis (ATN). These findings suggest that AKI in patients with KDSS results from ATN due to MODS. 

#### 3.2.5. Renal Acute Kidney Injury of Unknown Causes

Five patients with AKI of unknown causes have been reported. Rhodes et al. [56] reported an 11-year-old girl with KD who developed renal AKI and AHF due to myocarditis. She underwent mechanical ventilation, intravenous administrations of diuretics, and an intravenous administration of immunoglobulin (IVIG). The patient became well and recovered without any sequels. The authors suggested that AKI in the patient was due to nephritis. Yamawaki et al. [57] reported a 5-year-old boy with KD who developed renal AKI and underwent hemodialysis. The patient recovered completely without any renal sequels. Lande et al. [58] reported a 3-year-old girl with KD who developed renal AKI and underwent hemodialysis. An abdominal ultrasound revealed enlarged and echogenic kidneys. This patient also recovered without any renal sequels. El Karoui et al. [59] reported a 45-year-old man with KD who developed renal AKI, in whom IVIG resulted in a rapid improvement and recovery of normal renal function without any sequels. Nandi and Mondal [60] reported a 4-year-old boy with KD who developed renal AKI and recovered with supportive measures alone without requiring renal replacement therapy. 

## 4. Acute Nephritis Syndrome

ANS is the clinical picture of several glomerulonephropathies, characterized typically by hematuria, edema, hypertension, moderate proteinuria, and renal insufficiency [61, 62]. The ANS results from a reduction in the glomerular filtration rate caused by an inflammatory reaction in the glomeruli and is most often seen in patients with acute postinfectious glomerulonephritis [61]. Some forms of vasculitis also present with ANS, including Henoch-Schönlein purpura, Wegener's granulomatosis, and microscopic polyangiitis [62].

Seven cases of ANS have been reported in patients with KD [63–69]. Six patients were infants (median age, 5 months; range, 2–72 months). ANS developed between 2 and 30 days (median 20 days) following the onset of Kawasaki disease. Hematuria (macroscopic hematuria), proteinuria, edema, and hypertension occurred in 7/7 (5/7), 6/7, 5/7, and 4/7 of the patients, respectively. Decreased levels of both serums C3 and C4 were present in 4/7 of the patients. Mild renal insufficiency developed in two patients, which promptly resolved following the recovery of KD. The abnormal renal findings in all patients disappeared completely, while six patients developed CALs. A renal biopsy was only performed in a 3-year-old patient reported by Salcedo et al. [63] which revealed mesangioproliferative glomerulonephritis with interstitial infiltrates of lymphocytes and plasma cells by light microscopy, coarse granular deposition of IgM and C3 in the mesangium by immunofluorescent studies, and electron-dense deposits in the mesangium and in subendothelial space by electron microscopy.

The pathogenesis underlying the development of ANS in KD remains unclear. Immune-complex mediated mechanisms were suspected in some patients, because the renal biopsy findings of the patient reported by Salcedo et al. showed electron-dense deposits in the glomeruli, with 4 of the 7 patients having decreased levels of both serums C3 and C4, which suggested activation of the classical complement pathway. 

## 5. Nephrotic Syndrome

Although proteinuria is common in patients with KD, NS has been reported only rarely. Ogino et al. [70] reported a 3-year-old boy with KD who developed NS and azotemia, which resolved following the recovery of KD. Kidney and coronary angiographies showed no abnormal findings. Lee et al. [71] reported a 3-month-old boy with KD who developed NS. Although administration of corticosteroids improved NS in this patient, he died from a massive myocardial infarction on the 34th day of illness. Krug et al. [72] reported three children with KD, aged 4, 4.5, and 8 years, respectively, who developed NS. All three patients were treated with IVIG and aspirin, but none received corticosteroids. Proteinuria in all three patients disappeared within 2 weeks. Gatterre et al. [54] studied 11 patients with KDSS and reported that 3 of the 11 patients developed NS. Despite the severity of symptoms, all patients recovered from NS and survived without any sequels.

Because no renal biopsies were performed, the pathogenic mechanism underlying the development of NS in KD remains unclear. However, immune-complex mediated kidney injuries and/or T-cell immune-regulatory abnormalities similar to those seen in minimal change NS have been postulated as possible mechanisms of NS in KD [71].

 Joh et al. [73] reported a 4-month-old girl who developed NS 10 weeks following the onset of KD. The early renal histological lesion was similar to the Finnish-type congenital nephrotic syndrome at biopsy, but within 4 months the patient revealed diffuse mesangial sclerosis at autopsy. Because NS of the patient developed 10 weeks after the onset of KD, it seems likely that infantile NS in this patient may have been caused by abnormalities in genes not directly associated with KD such as *NPHS1*, *NPHS2*, *WT1,* or* LAMB2* [74]. 

## 6. Renal Tubular Abnormalities

Renal tubular abnormalities have sometimes been reported in patients with KD. Kondo et al. [75] studied urinary lysozyme and *β*2-microglobulin (*β*2MG) levels, parameters of renal tubular damage, in 16 patients with KD and reported that some patients with KD had elevated urinary lysozyme and *β*2MG levels during the acute phase. 

Asami et al. [76] examined urinary N-acetyl-*β*-D-glucosaminidase (NAG) levels, known as a sensitive index of renal tubular disorder, of six patients with KD and reported that all patients had elevated urinary NAG levels during the acute phase of the disease.

Ohta et al. [22] measured urinary interleukin-6 (IL-6), *β*2MG, and NAG levels of 20 patients with KD and showed that urinary IL-6, *β*2MG, and NAG levels were elevated in the majority of patients during the acute phase, indicating the presence of specific renal parenchymal lesions.

Igarashi et al. [77] examined urinary *β*2MG and NAG levels and serum *β*2MG concentrations in 12 patients with KD and showed that urinary *β*2MG and NAG levels were elevated in 10 of these patients during the acute phase, while no patients had elevated serum *β*2MG concentrations.

We studied urinary *β*2MG levels in 23 patients with KD [14]. These patients were divided into three groups according to the results of urinalysis: patients without pyuria, patients with pyuria in both voided urine and bladder urine obtained by transurethral catheterization (bladder pyuria), and patients with pyuria only in voided urine (urethral pyuria). We showed that the majority of patients with KD had elevated urinary *β*2MG levels, while patients with bladder pyuria had higher urinary *β*2MG levels than patients with urethral pyuria or patients without pyuria. These findings suggest that patients with KD have renal tubular abnormalities, especially patients with renal inflammation.

The pathogenesis of renal tubular abnormalities in KD remains unclear. Because KD patients with elevated urinary *β*2MG and NAG levels also have increased urinary IL-6 concentrations [22], the inflammatory process within the renal parenchyma, such as TIN may cause renal tubular abnormalities in these patients.

## 7. Renal Abnormalities in Imaging Studies

Renal abnormalities in imaging studies using renal ultrasonography (US) or technetium-99m dimercaptosuccinic acid scintigraphy single photon emission computed tomography (DMSA renal SPECT) have been reported in patients with KD.

Nardi et al. [78] examined seven patients with KD using renal US and reported that four patients with AKI had renal sonographic findings of increased cortical echogenicity, enlarged kidneys, and enhanced corticomedullary differentiation. They suggested that the enlargement of the kidneys may have been caused by a vasculitis involving the kidneys with the resulting fibrinoid deposits and cellular infiltrations leading subsequently to ischemia followed by edema. 

Huang et al. [79] measured the kidney lengths of 20 patients with KD and with normal renal function, 20 healthy children, and 15 febrile children using renal US. They also measured the levels of plasma hepatocyte growth factor (HGF) and transforming growth factor-*β*1 (TGF-*β*1) in all the children. The study showed that 14 (70%) of the patients with KD during the acute phase had absolute nephromegaly, with kidney lengths in these patients being significantly longer than those of healthy children or febrile children. In addition, the ratio of plasma HGF/TGF-*β*1 during the acute phase and after the recovery phase correlated positively with the degree of nephromegaly in all patients with KD. HGF is a potent mitogen and may act as a renotropic factor [80]. Increased serum levels of HGF have been reported in children with KD [81]. On the other hand, TGF-*β*1 is an inhibitor of cellular proliferation in many types of epithelial cells [80]. The reciprocal change between HGF and TGF-*β*1 may suggest a decreased antiproliferative effect of TGF-*β*1 on renal growth and also potentiate the mitogenic action of HGF, leading to nephromegaly in patients with KD.

Wang et al. [82] performed DMSA renal SPECT on 50 patients with KD and reported that 26 of these patients (52%) had renal inflammatory foci, in whom 11 of 24 patients (46%) still had renal scarring on a 6-month follow-up DMSA renal SPECT. These findings suggest that the potential long-term clinical impact of KD is not limited to CALs sequelae but also includes renal scar formation.

 Wu et al. [83] performed DMSA renal SPECT, renal Doppler ultrasonography to measure the pulsatility index (PI) and resistance index (RI), and analysis of urinary IL-6 levels in 50 patients with KD. Their study showed that 10 of 24 patients had renal inflammatory foci, that patients with renal inflammatory foci had significantly higher levels of urinary IL-6 and PI values than patients without renal inflammatory foci, and that there was a significant correlation between urinary IL-6 levels and PI values. These findings suggest that immune-mediated vasculitis is one of the mechanisms causing renal inflammation in KD.

## 8. Renal Artery Lesions 

Although CALs are a well-known vascular complication of KD, vasculitis has also been observed in extra-coronary artery including arteries in the kidney. In autopsy studies, panarteritis was observed frequently in the kidney [38, 39] and was localized in the interlobar arteries and occurred rarely in the arcuate and interlobular arteries [83]. Compared with coronary arteritis, arteritis in the kidney developed several days later and inflammation was milder [84].

 Renal artery aneurysms have been reported in patients with KD. Sasaguri and Kato [85] presented a patient with bilateral renal artery aneurysms without renovascular hypertension. Kato et al. [86] also reported that 5 of 594 patients with KD (0.8%) developed renal artery aneurysms. All patients with renal artery aneurysms in that report had coronary aneurysms, and no patient progressed to renovascular hypertension. 

 Renal artery stenosis has sometimes been reported in patients with KD, with or without renovascular hypertension. Negoro et al. [87] reported that an 11-month-old girl developed CALs, an abdominal aortic aneurysm and left renal artery stenosis 6 months after the onset of KD. Nagao and colleague [88] described a 3-year-old boy who developed right renal artery stenosis with renovascular hypertension 2 years following the acute phase of KD, which was successfully treated with percutaneous transluminal renal artery angioplasty (PTRA). Mawatari et al. [89] presented a 22-year-old man who developed renovascular hypertension due to right renal artery stenosis 14 years after the onset of KD, which was successfully treated with PTRA. Foster et al. [90] described an 18-month-old girl who developed renovascular hypertension due to right renal artery stenosis 6 months after the acute episode of KD. The patient underwent revascularization surgery because PTRA therapy could not conserve the right kidney function. Falcini et al. [91] reported two patients with KD (16-month-old girl and 3-month-old boy) who developed renovascular hypertension due to bilateral renal artery stenosis 3 weeks and 4 weeks after the onset of KD, respectively. 

In addition, there are two reports of patients with renovascular hypertension associated with abdominal aortic aneurysms caused by KD [92, 93]. 

## 9. Conclusions

Kidney and urinary involvement of KD is uncommon and includes the following conditions: pyuria, prerenal AKI, renal AKI caused by TIN, HUS, and immune-complex mediated nephropathy, renal AKI associated with KDSS or unknown causes, ANS, NS, renal tubular abnormalities, renal abnormalities in imaging studies, and renal artery lesions. Although the precise pathogenesis underlying the development of kidney and urinary tract involvement in patients with KD remains unclear, vasculitis of the arteries in the kidney, immune-complex mediated kidney injuries, and T-cell immune-regulatory abnormalities have been proposed as possible mechanisms.
